# Endothelial cell HSPA12B and yes-associated protein cooperatively regulate angiogenesis following myocardial infarction

**DOI:** 10.1172/jci.insight.139640

**Published:** 2020-09-17

**Authors:** Min Fan, Kun Yang, Xiaohui Wang, Yana Wang, Fei Tu, Tuanzhu Ha, Li Liu, David L. Williams, Chuanfu Li

**Affiliations:** 1 Department of Surgery and; 2 Center of Excellence in Inflammation, Infectious Disease and Immunity, James H. Quillen College of Medicine, East Tennessee State University (ETSU), Johnson City, Tennessee, USA.; 3 Department of Developmental Biology, Harvard School of Dental Medicine, Boston, Massachusetts, USA.; 4 Department of Geriatrics, The First Affiliated Hospital of Nanjing Medical University, Nanjing, China.

**Keywords:** Angiogenesis, Vascular Biology, Cardiovascular disease, endothelial cells, hypoxia

## Abstract

Angiogenesis is essential for cardiac functional recovery after myocardial infarction (MI). HSPA12B is predominately expressed in endothelial cells and required for angiogenesis. Yes-associated protein (YAP) plays an important role in tumor angiogenesis. This study investigated the cooperative role of HSPA12B and YAP in angiogenesis after MI. Silencing of either HSPA12B or YAP impaired hypoxia-promoted endothelial cell proliferation and angiogenesis. Deficiency of HSPA12B suppressed YAP expression and nuclear translocation after hypoxia. Knockdown of YAP attenuated hypoxia-stimulated HSPA12B nuclear translocation and abrogated HSPA12B-promoted endothelial cell angiogenesis. Mechanistically, hypoxia induced an interaction between endothelial HSPA12B and YAP. ChIP assay showed that HSPA12B is a target gene of YAP/transcriptional enhanced associated domain 4 (TEAD4) and a coactivator in YAP-associated angiogenesis. In vivo studies using the MI model showed that endothelial cell–specific deficiency of HSPA12B (e*Hspa12b*^–/–^) or YAP (e*Yap*^–/–^) impaired angiogenesis and exacerbated cardiac dysfunction compared with WT mice. MI increased YAP expression and nuclear translocation in WT hearts but not e*Hspa12b*^–/–^ hearts. HSPA12B expression and nuclear translocation were upregulated in WT MI hearts but not e*Yap*^–/–^ MI myocardium. Our data demonstrate that endothelial HSPA12B is a target and coactivator for YAP/TEAD4 and cooperates with YAP to regulate endothelial angiogenesis after MI.

## Abstract 2

Endothelial HSPA12B is a target and co-activator for YAP/TEAD4 and cooperates with YAP to regulate endothelial angiogenesis post-MI.

## Graphical abstract

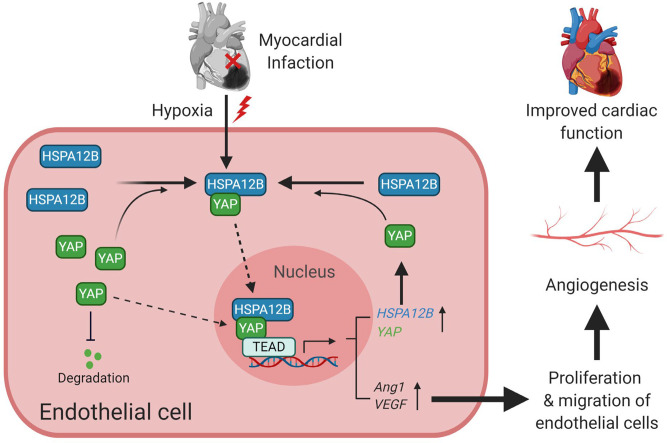

## Introduction

Heart failure after myocardial infarction (MI) remains the leading cause of mortality among all cardiovascular diseases globally (1, 2). Angiogenesis plays a critical role in the prevention of the development of heart failure by improving left ventricular remodeling after MI (3, 4). Recent studies have shown that stimulation of endothelial cells to the proliferative and migratory phenotypes are essential for angiogenesis (3–7).

Heat shock protein (HSP) A12B (HSPA12B) was initially discovered from atherosclerotic lesions as a member of the HSP70 family (8). HSPA12B is mainly expressed in vascular endothelium and is required for angiogenesis in both zebrafish and human umbilical vein endothelial cells (HUVECs) (9, 10). Subsequent studies showed that increased expression of HSPA12B attenuated cardiac dysfunction and improved ventricular remodeling after MI or endotoxin challenge (11, 12). Our previous studies demonstrated that HSPA12B protects against cerebral ischemia/reperfusion (I/R) injury and inhibits LPS-induced inflammatory response in HUVECs through activation of PI3K/Akt signaling pathway (13, 14). Additionally, HSPA12B promotes angiogenesis after ischemic stroke through eNOS-dependent signaling (15). However, the detailed cellular and molecular mechanisms by which HSPA12B regulates angiogenesis after MI have not been elucidated. In addition, the mechanisms by which MI injury or hypoxia upregulates HSPA12B production remain unclear.

Yes-associated protein (YAP) is a key effector in Hippo/YAP signaling and plays an important role in organ size control and tumorigenesis by regulating cell proliferation and apoptosis (16–19). Cardiac-specific activation of YAP has been reported to improve cardiac function and survival rate after MI (20). In contrast, cardiac-specific deletion of YAP impaired cardiac regeneration after MI at least partly through regulating IGF1 and Akt signaling (21). We have previously reported that YAP is required for neonatal heart regeneration after cardiac I/R (22). We demonstrated that TLR3-mediated glycolysis inhibits activation of large tumor suppressor kinase 1 (LATS1) and AMPK, leading to YAP activation, which promotes neonatal cardiomyocyte proliferation through miR-152. In the embryonic stage, direct knockout of cardiomyocyte-specific YAP shows insufficient cardiomyocyte proliferation, leading to fatal cardiac hypoplasia (23, 24). Activation of YAP promotes both fetal and neonatal cardiomyocyte proliferation via interaction of YAP and transcriptional enhanced associated domain (TEAD) (23) and activation of IGF-dependent PI3K/Akt signaling (24). In addition, several studies have shown that YAP plays an important role in angiogenesis for tumor growth (25, 26). In addition, endothelial cell YAP/TAZ is required for vascular network formation in the brain (27). However, the role of YAP in angiogenic remodeling after MI and the underlying mechanisms remains elusive.

The present study investigated whether endothelial cell–specific HSPA12B and YAP cooperatively regulated angiogenesis after MI. We observed that hypoxia-promoted angiogenesis was associated with HSPA12B nuclear translocation and concomitant YAP activation and translocation into nucleus. We demonstrated that endothelial cell HSPA12B was a target gene of YAP and a coactivator in YAP-associated angiogenesis. Our data indicate that endothelial cell HSPA12B and YAP cooperatively regulated angiogenesis after MI.

## Results

### YAP activation was required for HSPA12B-promoted endothelial cell proliferation, migration, and angiogenesis after hypoxia.

**Figure 1:**
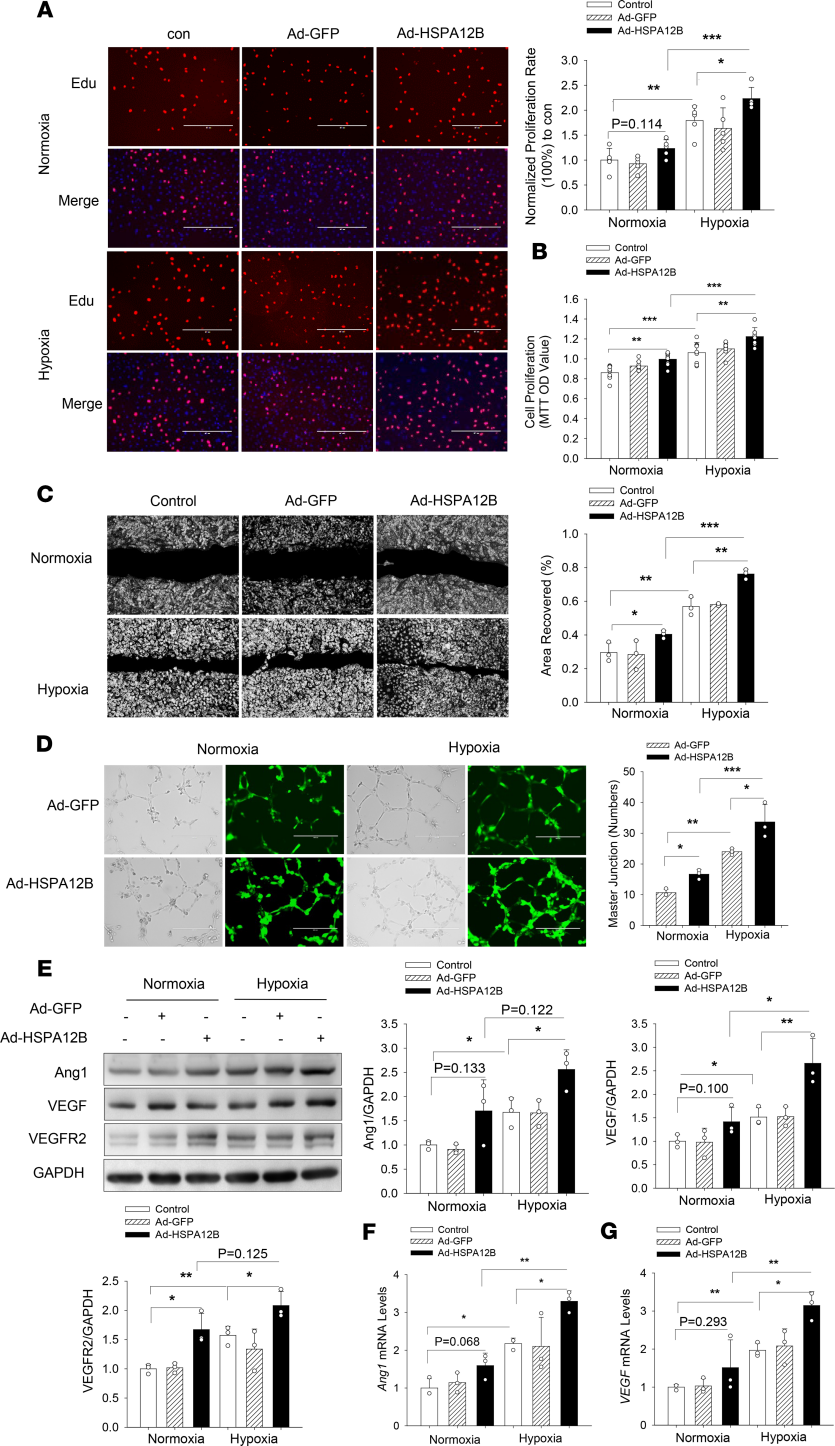
HSPA12B promotes endothelial cell proliferation, migration, and angiogenesis after hypoxic challenge. HUVECs were transfected with adenovirus expressing HSPA12B (Ad-HSPA12B) or Ad-GFP. Twenty-four hours after transfection, cells were subjected to hypoxia or normoxia. Cell proliferation was examined by Edu incorporation (**A**) and MTT assay (**B**) (scale bar: 400 μm). (**C**) Cell migration was examined by wound-healing assay (scale bar: 400 μm). (**D**) Angiogenesis was examined by Matrigel assay (scale bar: 400 μm). The levels of angiogenetic markers (Ang1, VEGF, and VEGFR2) were examined by Western blot (**E**) and qRT-PCR (**F** and **G**). GAPDH was used as loading control. *n* = 3 independent experiments/group. Comparisons of data between groups were made using 1-way ANOVA followed by Tukey’s procedure. *P < 0.05, **P < 0.01, ***P < 0.001 compared with indicated groups. HUVECs, human umbilical vein endothelial cells; Edu, 5-ethynyl-2-deoxyuridine.

Transgenic mice with endothelial cell–specific overexpression of HSPA12B promote angiogenesis after MI (11). To validate whether endothelial HSPA12B could be an effector for angiogenesis, we transfected HUVECs with adenoviral HSPA12B (Ad-HSPA12B) or Ad-GFP 24 hours before the cells were subjected to hypoxia. Endothelial cell proliferation was measured by 5-ethynyl-2-deoxyuridine (Edu) incorporation and MTT assay. As shown in Figure 1, A and B, hypoxia alone increased endothelial cell proliferation. Increased expression of HSPA12B by Ad-HSPA12B transfection further promoted hypoxia-induced cell proliferation by 24.6% (Edu incorporation) and 15.2% (MTT assay) compared with respective hypoxic controls. In addition, increased expression of HSPA12B also profoundly increased hypoxia-induced endothelial cell migration by 36.7% compared with hypoxic control (Figure 1C). The Matrigel-based angiogenesis assay showed that Ad-HSPA12B transfection significantly increased tube formation in both normoxia and hypoxia groups (Figure 1D).

**Figure 2:**
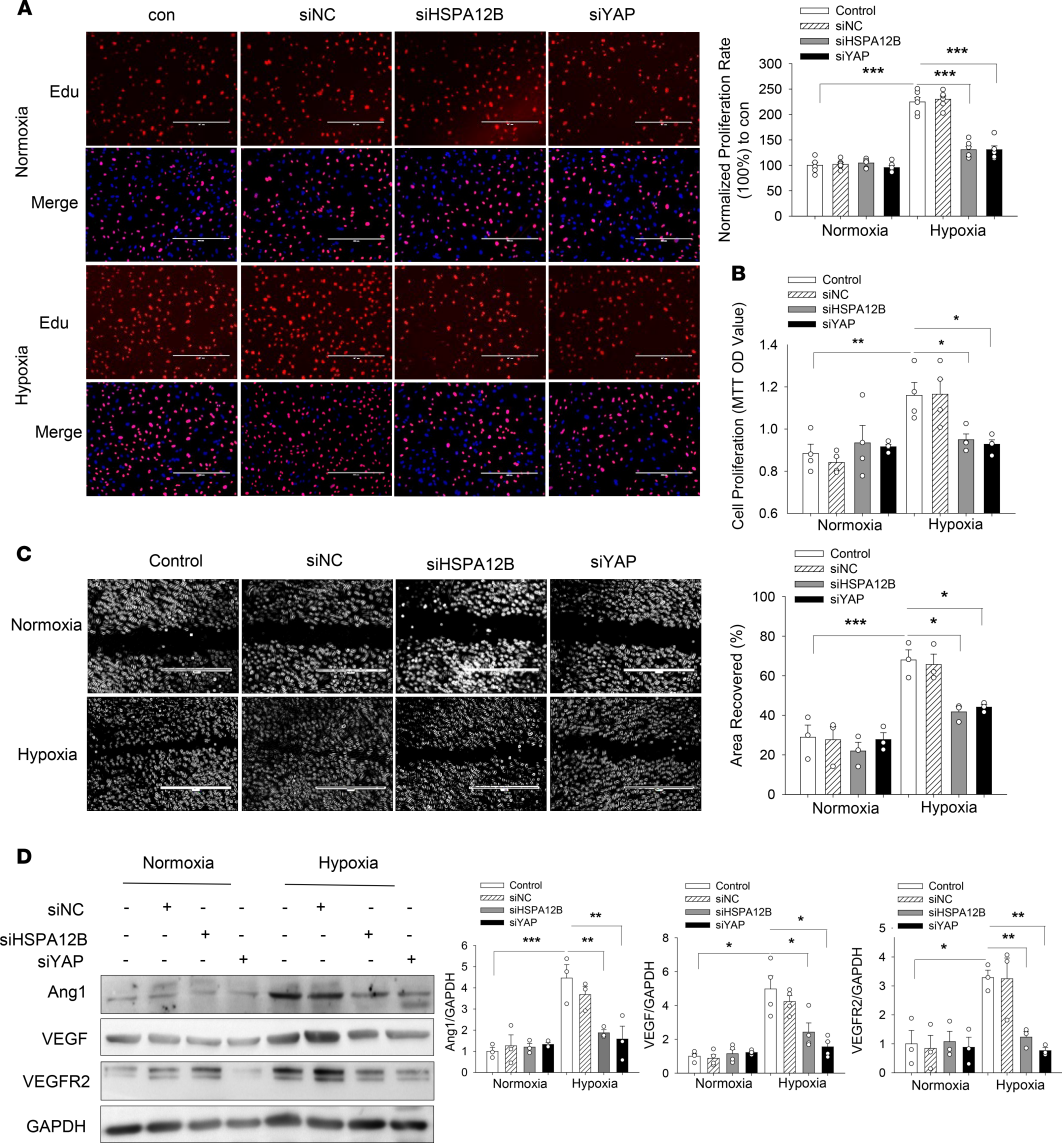
siRNA silencing of HSPA12B or YAP attenuates hypoxia-induced endothelial cell proliferation, migration, and angiogenesis. HUVECs were transfected with siRNA specific for HSPA12B (siHSPA12B) or for YAP (siYAP). Scrambled siRNA served as control (siNC). Twenty-four hours after transfection, the cells were subjected to hypoxia or normoxia. Cell proliferation was measured by Edu incorporation (*n* = 3) (**A**) and MTT assay (*n* = 4) (**B**) (scale bar: 400 μm). (**C**) Cell migration was examined by wound-healing assay (*n* = 3) (scale bar: 1000 μm). (**D**) The levels of Ang1 (*n* = 3), VEGF (*n* = 4), and VEGFR2 (*n* = 3) were examined by Western blot. GAPDH was used as loading control. Comparisons of data between groups were made using 1-way ANOVA followed by Tukey’s procedure. *P < 0.05, **P < 0.01, ***P < 0.001 compared with indicated groups. HUVECs, human umbilical vein endothelial cells; YAP, yes-associated protein; Edu, 5-ethynyl-2-deoxyuridine.

Angiogenetic factors such as VEGF, angiopoietin-1 (Ang1), and VEGFR2 play a critical role in the regulation of endothelial cell proliferation and angiogenesis (28–32). Therefore, we examined the effect of HSPA12B on the expression of VEGF, Ang1, and VEGFR2 after hypoxic challenge. Figure 1E shows that hypoxic challenge upregulated expression of Ang1, VEGF, and VEGFR2 in HUVECs. Importantly, transfection of HUVECs with Ad-HSPA12B further increased the levels of Ang1, VEGF, and VEGFR2 compared with hypoxia control. In addition, transfection of HUVECs with Ad-HSPA12B markedly increased the mRNA levels of *Ang1* and *VEGF* (Figure 1, F and G). To confirm the role of HSPA12B in hypoxia-induced endothelial cell proliferation and angiogenesis, we reduced HSPA12B expression by its specific siRNA before subjecting endothelial cells to hypoxic challenge. Silencing of HSPA12B expression markedly suppressed hypoxia-induced endothelial cell proliferation and migration (Figure 2, A–C). Moreover, Figure 2D shows that silencing of HSPA12B markedly suppressed hypoxia-induced expression of angiogenetic factors. Collectively, our data suggest that HSPA12B played an important role in upregulating angiogenetic factor expression, which promotes endothelial cell proliferation, migration, and angiogenesis.

**Figure 3:**
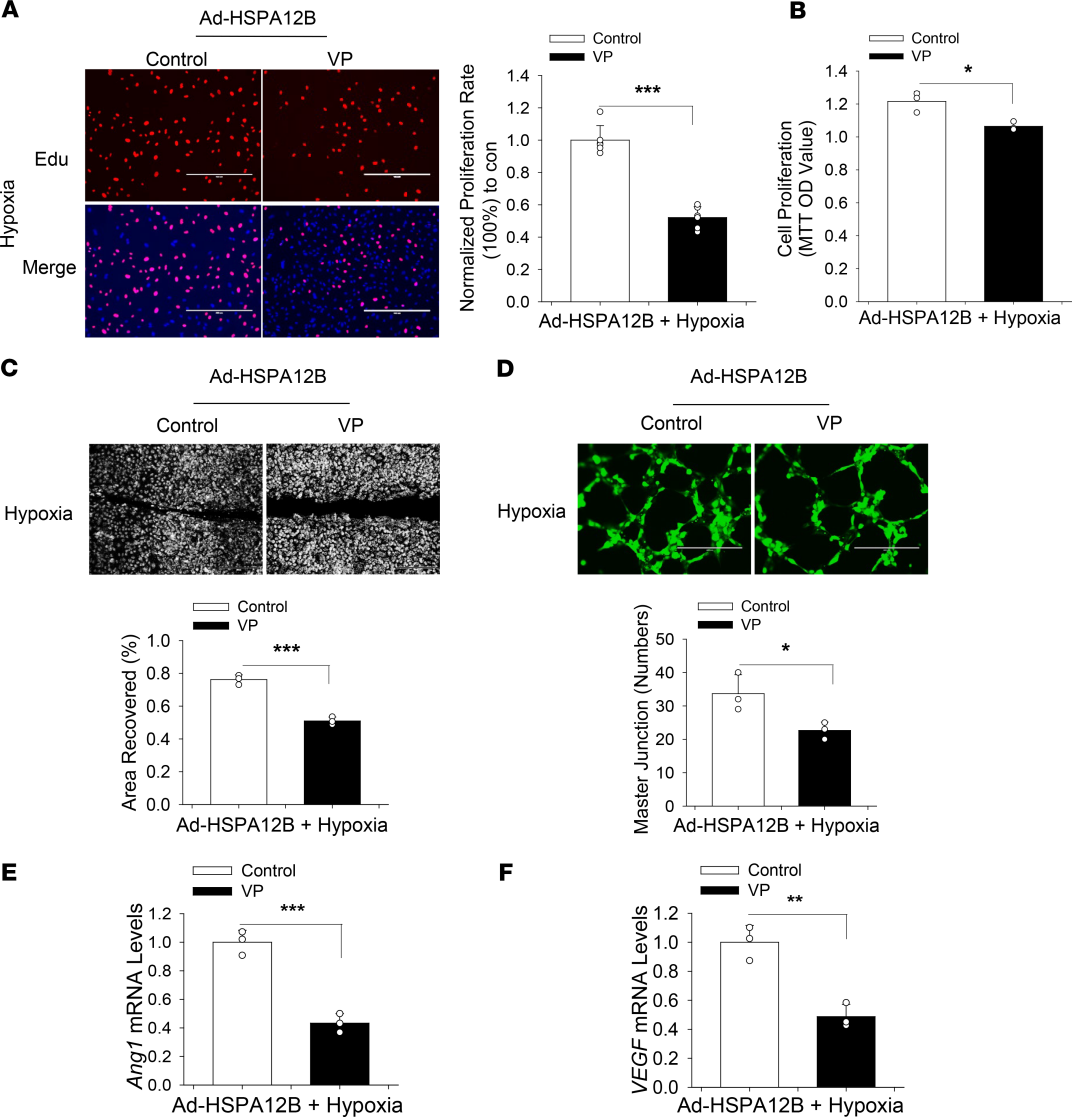
YAP inhibition suppresses HSPA12B-induced endothelial cell proliferation, migration, and angiogenesis after hypoxia. HUVECs were transfected with Ad-HSPA12B or Ad-GFP 24 hours before the cells were treated with YAP inhibitor, VP (1 Mm/L). The cells were then subjected to hypoxia or normoxia. Cell proliferation was evaluated by Edu incorporation (**A**) and MTT assay (**B**) (scale bar: 400 μm). (**C**) Cell migration was examined by wound-healing assay (scale bar: 400 μm). (**D**) Angiogenesis was examined by Matrigel assay (scale bar: 400 μm). The mRNA levels of *Ang1* and *VEGF* were examined by qRT-PCR (**E** and **F**). *n* = 3 independent experiments/group. Comparisons of data between groups were made using 2-tailed *t* test. *P < 0.05, ^**^P < 0.01, ^***^P < 0.001 compared with indicated groups. HUVECs, human umbilical vein endothelial cells; YAP, yes-associated protein; Edu, 5-ethynyl-2-deoxyuridine.

Previous studies show several HSPs, including HSP90 and HSP27, are involved in regulation of the Hippo/YAP pathways (33, 34). Furthermore, substantial evidence associates YAP with cell proliferation, migration, and angiogenesis in multiple organisms (16–18, 26, 27). We next sought to investigate whether YAP activation could be reconciled with HSPA12B-promoted angiogenesis after hypoxia. To this end, endothelial cells were transfected with Ad-HSPA12B 24 hours before the cells were treated with YAP inhibitor verteporfin (VP) (35) followed by hypoxia. Edu incorporation, MTT assay, and the wound-healing assay showed that YAP inhibition attenuated overexpression of HSPA12B-promoted cell proliferation (Figure 3, A and B), migration (Figure 3C), and angiogenesis (Figure 3D). Quantitative real-time PCR (qRT-PCR) data show that YAP inhibition markedly attenuated overexpression of HSPA12B-promoted expression of *Ang1* mRNA levels by 56.7% and *VEGF* mRNA levels by 51.3% (Figure 3, E and F) compared with the control group. Together, our results provide evidence that YAP was required for HSPA12B-promoted proliferation, migration, and angiogenesis after hypoxic challenge.

### HSPA12B was required for YAP expression and nuclear translocation in endothelial cells after hypoxic challenge.

YAP plays an important role in angiogenesis during tumor growth (25, 26). We further examined the regulatory role of YAP on hypoxia-induced endothelial cell proliferation, migration, and angiogenesis. We first reduced YAP expression in HUVECs by siRNA transfection before hypoxic challenge. As shown in Figure 2, A–D, silencing of YAP prevented hypoxia-induced cell proliferation, migration, and expression of angiogenetic factors (VEGF, Ang1, and VEGFR2). In parallel, deactivation of YAP by VP suppressed hypoxia-stimulated cell proliferation, migration, and angiogenesis (Supplemental Figure 1, A–D; supplemental material available online with this article; https://doi.org/10.1172/jci.insight.139640DS1) and attenuated hypoxia-induced expression of *Ang1* and *VEGF* (Supplemental Figure 1, E–G), indicating that YAP was involved in hypoxia-induced endothelial cell proliferation and angiogenesis.

**Figure 4:**
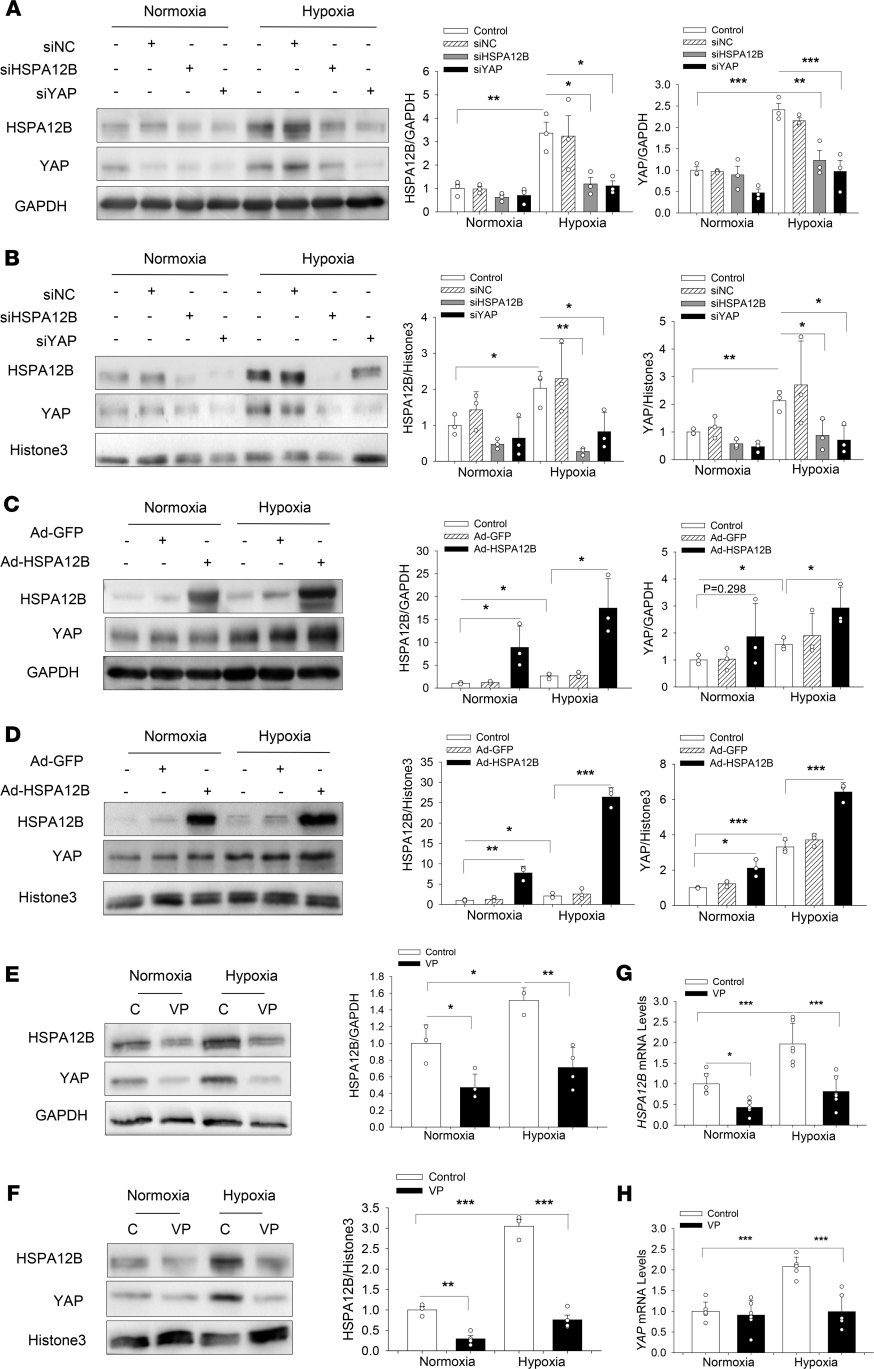
Cooperative regulation of HSPA12B and YAP expression and nuclear localization in endothelial cells after hypoxic challenge. HUVECs were transfected with siRNA specific for HSPA12B (siHSPA12B) or for YAP (siYAP). Scrambled siRNA served as control (siNC). In separate experiments, HUVECs were transfected with Ad-HSPA12B or Ad-GFP. Twenty-four hours after transfection, cells were subjected to hypoxia or normoxia. The levels of HSPA12B and YAP in the cytosol (**A** and **C**) and the nucleus (**B** and **D**) were examined by Western blot (*n* = 3). (**E–H**) Endothelial cells were treated with YAP inhibitor, verteporfin (1 Mm), before the cells were subjected to hypoxia. The levels of HSPA12B and YAP in the cytosol and the nucleus were examined by Western blot (*n* = 3–4, **E** and **F**). GAPDH was used as cytosolic loading control and Histone3 was used as nuclear loading control. The mRNA levels of *HSPA12B* and *YAP* were assessed with qRT-PCR (*n* = 3) (**G** and **H**). Comparisons of data between groups were made using 1-way ANOVA followed by Tukey’s procedure. *P < 0.05, ^**^P < 0.01, ^***^P < 0.001 compared with indicated groups. HUVECs, human umbilical vein endothelial cells; YAP, yes-associated protein.

To gain insights into whether HSPA12B was involved in YAP-promoted endothelial cell angiogenesis after hypoxia, we examined the expressions of both YAP and HSPA12B in endothelial cells after hypoxia and found that hypoxia alone markedly induced both HSPA12B and YAP expression and nuclear translocation (Figure 4, A and B). However, silencing of HSPA12B with siRNA dramatically suppressed hypoxia-induced YAP expression and nuclear translocation (Figure 4, A and B). In contrast, increased expression of HSPA12B in HUVECs by transfection with Ad-HSPA12B significantly increased YAP expression and nuclear translocation after hypoxic challenge (Figure 4, C and D) compared with control group. Taken together, these data suggest that HSPA12B was required for YAP expression and nuclear translocation after hypoxic challenge. Of note, trafficking and accumulation of HSPA12B in the nucleus has not been previously observed, suggesting that HSPA12B may have an important yet unknown function in the nucleus during hypoxia (Figure 4B).

### YAP regulated HSPA12B expression and nuclear translocation in endothelial cells after hypoxia.

To explore whether YAP was required for hypoxia-promoted HSPA12B expression and nuclear translocation, we silenced YAP expression by siRNA transfection and examined HSPA12B expression in both cytoplasm and nucleus after hypoxic challenge. As shown in Figure 4, A and B, reduced YAP expression partially blocked hypoxia-promoted HSPA12B expression and nuclear translocation. Consistently, deactivation of YAP by VP administration also suppressed HSPA12B expression and nuclear translocation in the endothelial cells challenged with hypoxia (Figure 4, E and F). qRT-PCR analysis shows that YAP inhibition also suppressed hypoxia-induced increase in the levels of *HSPA12B* and *YAP* mRNA (Figure 4, G and H). These data demonstrate that YAP was involved in the regulation of HSPA12B expression and nuclear translocation after hypoxic challenge.

### Hypoxia induced an interaction between YAP and HSPA12B.

**Figure 5:**
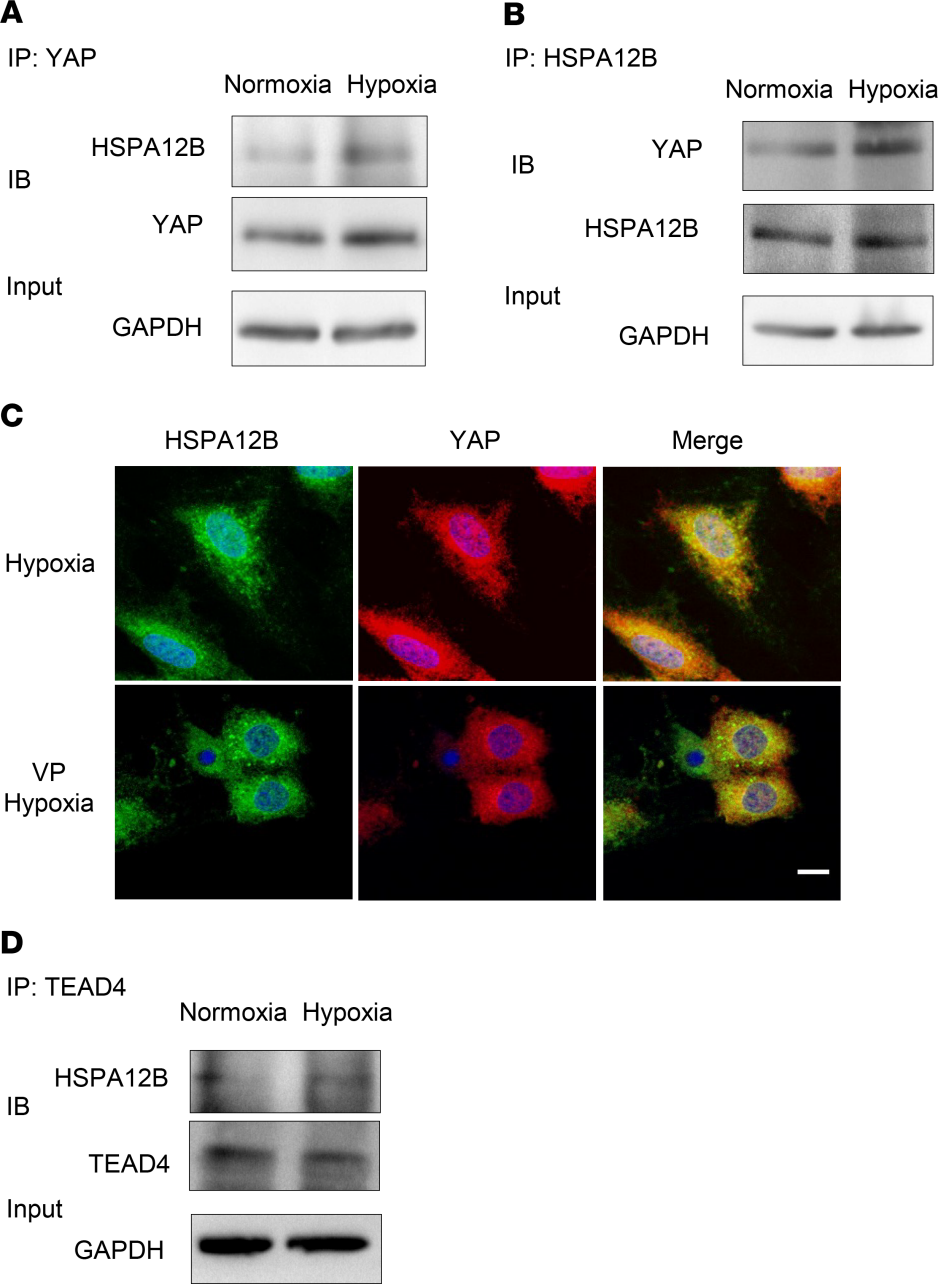
HSPA12B interacts with YAP and TEAD4 in endothelial cells after hypoxic challenge. HUVECs were subjected to hypoxia or normoxia for 24 hours. Cells were harvested for the isolation of cellular proteins. (**A**) Immunoprecipitation was performed with anti-YAP antibody followed by immunoblot with anti-HSPA12B antibody (*n* = 2). (**B**) Immunoprecipitation was performed with anti-HSPA12B antibody followed by immunoblot with anti-YAP antibody (*n* = 2). GAPDH was used as loading control. (**C**) HUVECs were treated with or without VP (1 Mm) before subjected to hypoxia. Immunostaining was performed with anti-HSPA12B (green) and anti-YAP (red) antibodies (*n* = 2) (scale bar: 10 μm). (**D**) Immunoprecipitation was performed with anti-TEAD4 antibody followed by immunoblot with anti-HSPA12B antibody (*n* = 2). GAPDH was used as loading control. HUVECs, human umbilical vein endothelial cells; YAP, yes-associated protein.

We then investigated whether there was a biologically cooperative interaction between YAP and HSPA12B in regulating endothelial cell proliferation and angiogenesis during hypoxia. HUVECs were subjected to hypoxia or normoxia, which served as control. Cellular proteins were isolated for immunoprecipitation with anti-YAP antibody followed by immunoblot with anti-HSPA12B antibody. Figure 5A shows that hypoxia significantly induced an interaction between YAP and HSPA12B, as evidenced by HSPA12B appearing in the immunoprecipitate with anti-YAP antibody. We also performed the immunoprecipitation with anti-HSPA12B antibody followed by immunoblotting with anti-YAP antibody. Reciprocally, YAP appeared in the immunoprecipitate with anti-HSPA12B antibody (Figure 5B). To further validate this observation, immunofluorescence staining was performed with anti-YAP (red color) and HSPA12B (green color) antibodies. As shown in Figure 5C, there was significant cytoplasmic and nuclear colocalization of YAP and HSPA12B after hypoxic challenge. However, inhibition of YAP by VP suppressed YAP nuclear localization and blocked the nuclear location of YAP with HSPA12B.

### HSPA12B is a YAP/TEAD4 target in HUVECs.

**Figure 6:**
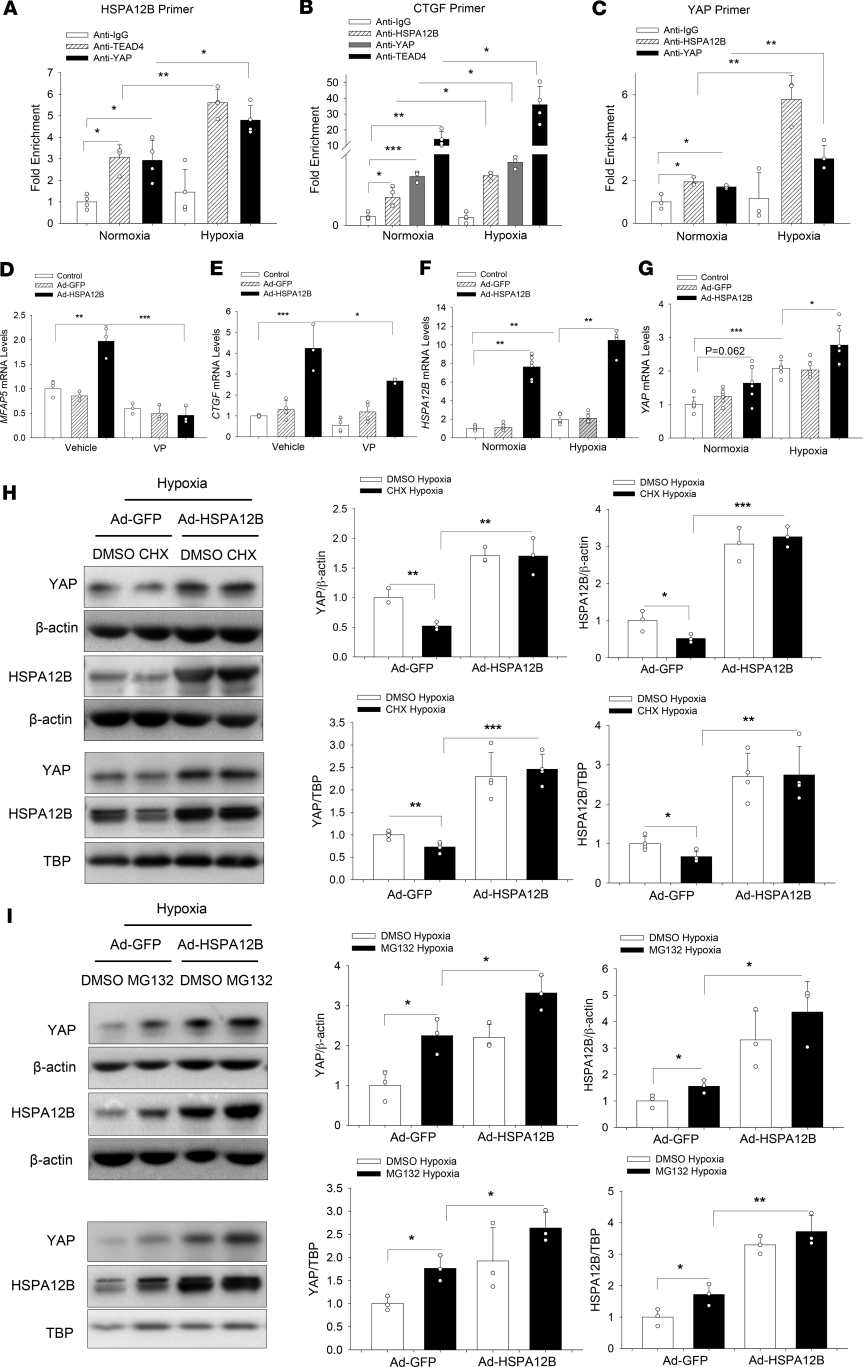
HSPA12B is a target and coeffector of YAP in endothelial cells. HUVECs were subjected to hypoxia or normoxia for 24 hours. ChIP in HUVECs with indicated antibodies (HSPA12B, YAP, or TEAD4) followed by qPCR using primers specific for indicated regions of HSPA12B (*n* = 4) (**A**), CTGF (*n* = 4) (**B**), and YAP (*n* = 3) (**C**). HUVECs were transfected with Ad-HSPA12B or Ad-GFP. Twenty-four hours after transfection, cells were subjected to hypoxia or normoxia. The mRNA levels of *HSPA12B* (*n* = 3) (**F**) and *YAP* (*n* = 3) (**G**) were examined by qRT-PCR. HUVECs were treated with or without verteporfin (1 Mm) before hypoxia. The mRNA levels of *MFAP5* (*n* = 3) (**D**) and *CTGF* (*n* = 3–4) (**E**) were examined by qRT-PCR. (**H** and **I**) Cells were treated with or without cycloheximide (100 μg/mL) or MG132 (12 μM) before hypoxia. The levels of HSPA12B and YAP in the cytosol and the nucleus were examined by Western blot (*n* = 3–4). β-actin was used as cytosolic loading control and TBP was used as nuclear loading control. Comparisons of data between groups were made using 1-way ANOVA followed by Tukey’s procedure. *P < 0.05, ^**^P < 0.01, ^***^P < 0.001 compared with indicated groups. HUVECs, human umbilical vein endothelial cells; YAP, yes-associated protein.

Because we observed that YAP inhibition decreased HSPA12B mRNA levels (Figure 4G), we next investigated whether HSPA12B was a direct transcriptional target of YAP in HUVECs. YAP controls gene transcription via binding to TEAD around the promoter or enhancer of target genes (36, 37). Therefore, we searched for and identified TEAD4-binding motif in the *HSPA12B* gene-enhancer regions. ChIP was performed to validate the potential binding sites of YAP/TEAD4 protein in the *HSPA12B* gene-enhancer region. As shown in Figure 6A, ChIP assay using anti-YAP or anti-TEAD4 indicated that both YAP and TEAD4 specifically associated with the enhancer region of *HSPA12B* gene, which can be induced by hypoxia. These data suggest that HSPA12B was a YAP/TEAD4 direct target in HUVECs.

### HSPA12B was a coactivator in YAP/TEAD4-regulated angiogenesis.

We observed that HSPA12B could translocate into the nucleus and that immunoprecipitation showed an interaction between HSPA12B and YAP/TEAD4, which can be induced by hypoxic challenge (Figure 5D). To determine whether nuclear HSPA12B could act as a coactivator of YAP/TEAD4 complex to regulate target gene transcription, we first examined the mRNA levels of YAP/TEAD4 target genes *MFAP5* and *CTGF*, which are related to angiogenesis and proliferation, respectively (26). As expected, the mRNA expression of *MFAP5* and *CTGF* was enhanced remarkably by overexpression of HSPA12B alone, which can be abolished by YAP inhibitor VP administration (Figure 6, D and E). ChIP assay also reveals that HSPA12B colocalized with YAP and TEAD4 at the promoter region of CTGF (Figure 6B). These data indicate that HSPA12B and YAP/TEAD4 formed a complex that synergistically activated target genes involved in cellular proliferation and angiogenesis.

To better understand the underlying molecular mechanism of HSPA12B-mediated YAP regulation, we examined the possibility of HSPA12B protein with *YAP* gene regulatory elements. Surprisingly, we detected a binding activity of HSPA12B protein and *YAP* gene enhancer (Figure 6C). In parallel, qPCR data show that increased expression of HSPA12B by Ad-HSPA12B transfection markedly upregulated *YAP* mRNA levels induced by hypoxia (Figure 6, F and G), indicating that HSPA12B serves as a transcriptional coactivator in regulating YAP expression. Furthermore, we treated endothelial cells with cycloheximide (CHX), a protein translation inhibitor (38), and observed that CHX administration significantly decreased HSPA12B and YAP cytosolic and nuclear expression after hypoxic challenge (Figure 6H). However, transfection with Ad-HSPA1B reversed YAP expression and nuclear translocation, indicating that upregulation of HSPA12B stabilizes YAP protein. In addition, administration of MG132, a proteasome inhibitor (39), induced YAP cytosolic and nuclear expression after hypoxia, whereas overexpression of HSPA12B by Ad-HSPA12B transfection further increased MG132-induced YAP expression and nuclear translocation (Figure 6I), which showed that HSPA12B overexpression promoted YAP transcription. Together, our data demonstrate that binding to HSPA12B stabilized YAP protein and prevented YAP from degradation. Moreover, HSPA12B functioned as a coactivator in YAP/TEAD4-regulated gene transcription during hypoxia-induced angiogenesis.

### Endothelial cell HSPA12B deficiency impaired angiogenesis and decreased YAP expression in the myocardium after MI.

**Figure 7:**
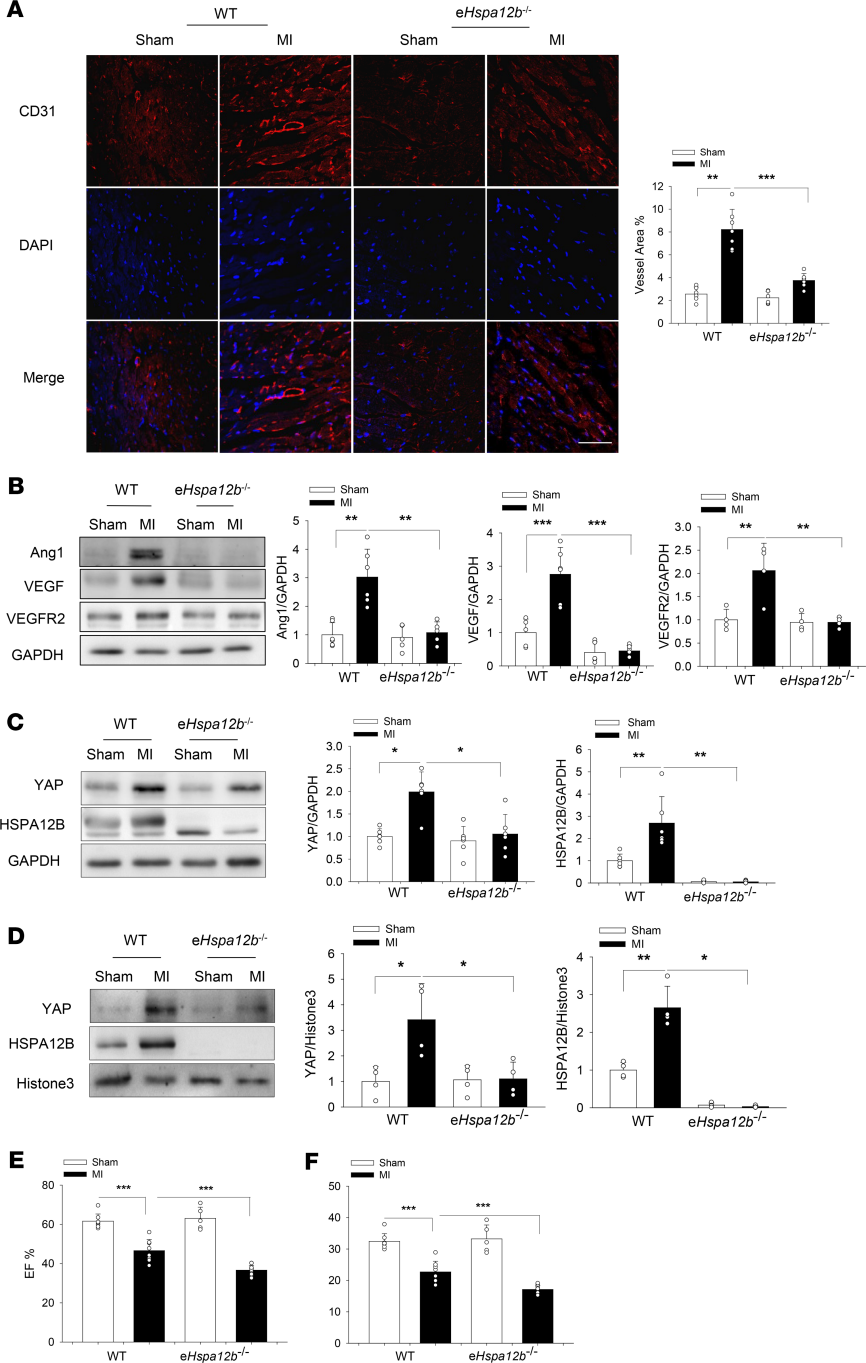
Endothelial *Hspa12b* deficiency worsens cardiac dysfunction, impairs angiogenesis, and decreases YAP expression and nuclear translocation after MI. WT and endothelial cell–specific Hspa12b-knockout (e*Hspa12b*^–/–^) mice were subjected to myocardial infarction (MI) or sham surgical operation. (**A**) Cardiac angiogenesis was examined by immunostaining of heart tissue sections with specific anti-CD31 antibody (*n* = 6–7) (scale bar: 100 μm). (**B**) The levels of angiogenetic markers Ang1 (*n* = 5–6), VEGF (*n* = 5–6), and VEGFR2 (*n* = 4) in the myocardium were examined by Western blot. The levels of HSPA12B and YAP in the cytosol (*n* = 5–6) (**C**) and nucleus (*n* = 4) (**D**) of the myocardium were examined by Western blot. GAPDH was used as loading control. Cardiac function was examined by echocardiography 28 days after surgery among WT sham (*n* = 8), WT MI (*n* = 8), e*Hspa12b*^–/–^ sham (*n* = 5), and eHSPA12B^–/–^ MI (*n* = 12) groups. (**E**) Ejection fraction (EF%). (**F**) Fractional shortening (FS%). Comparisons of data between groups were made using 1-way ANOVA followed by Tukey’s procedure. *P < 0.05, ^**^P < 0.01, ^***^P < 0.001 compared with indicated groups. YAP, yes-associated protein.

Our in vitro data suggest that endothelial cell HSPA12B was a coactivator for YAP regulating the expression of gene associated with angiogenesis and that HSPA12B was a target of YAP/TEAD4. We then examined the effect of endothelial cell–specific deficiency of *Hspa12b* (e*Hspa12b*^–/–^) on cardiac angiogenesis and YAP expression after MI. Cardiac angiogenesis was evaluated by immunofluorescence staining of cluster of differentiation 31 (CD31) in the myocardium 4 weeks after MI. As shown in Figure 7A, there were more positive immunofluorescence staining of CD31 in WT MI myocardium compared with WT sham controls. However, the CD31-positive immunofluorescence staining in e*Hspa12b*^–/–^ MI hearts was markedly reduced by 54.4% compared with WT MI hearts. The levels of VEGF, Ang1, and VEGFR2 in the *eHspa12b^–/–^* MI hearts were also significantly lower than those in WT MI myocardium (Figure 7B). Collectively, these data suggest that endothelial HSPA12B played an essential role in the regulation of angiogenesis after MI.

We next sought to investigate whether YAP expression could be reconciled with HSPA12B-promoted angiogenesis after MI. Figure 7, C and D, shows that the cytosolic and nuclear YAP levels of WT MI hearts, but not *eHspa12b^–/–^* MI myocardium, were significantly increased compared with sham controls. Interestingly, cytosolic and nuclear HSPA12B levels from WT hearts were also markedly increased (112.7%) after MI challenge, which was consistent with the data observed in endothelial cells (Figure 4, A and B). Immunofluorescence staining of HSPA12B validated the nuclear translocation of HSPA12B after MI (Supplemental Figure 2). The data suggest that endothelial HSPA12B can be translocated into the nucleus and that endothelial HSPA12B was required for YAP activation and nuclear translocation in the response to MI challenge.

### Endothelial cell YAP depletion impaired angiogenesis and decreased HSPA12B expression in the myocardium after MI.

**Figure 8:**
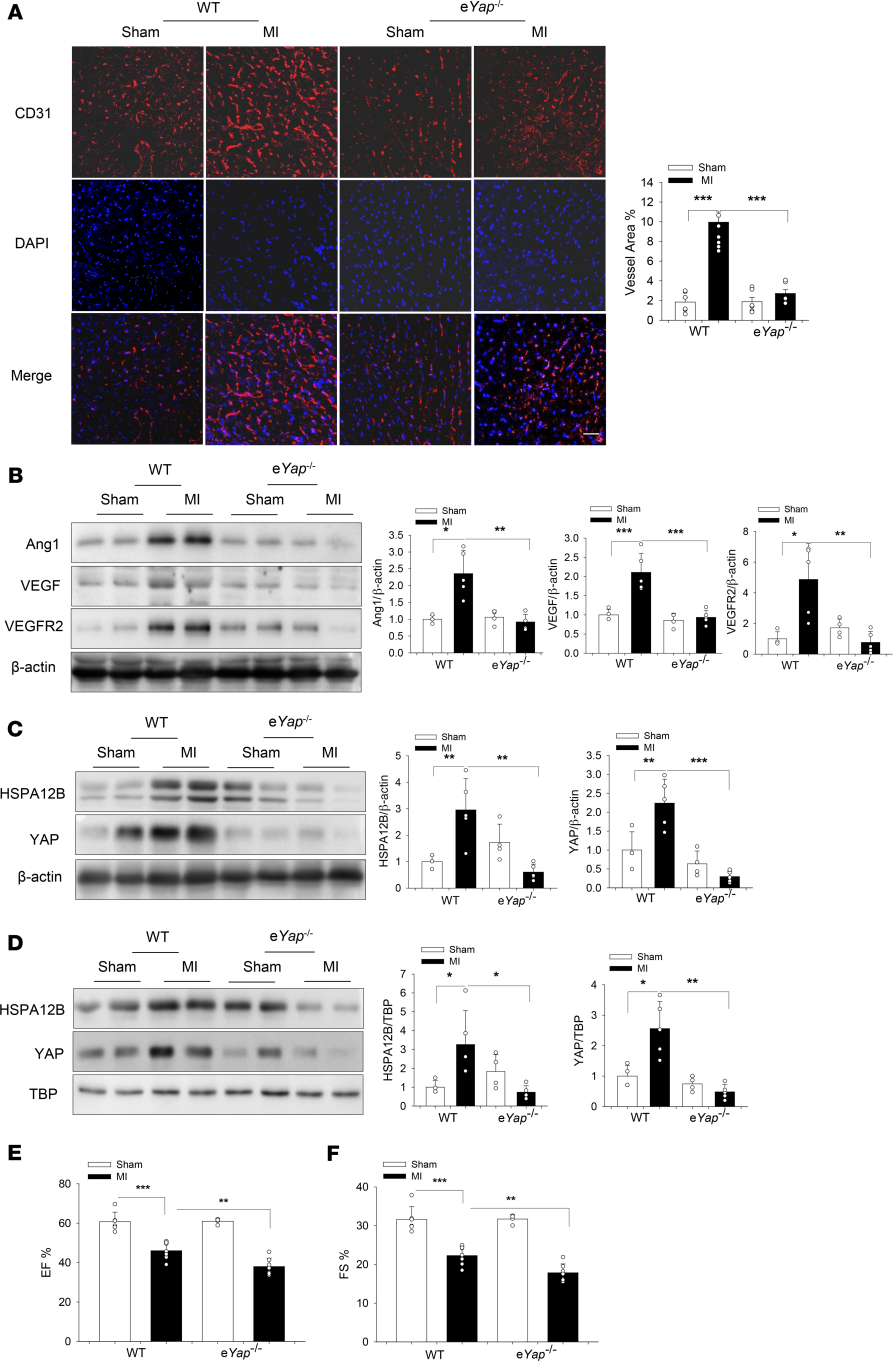
Endothelial cell–specific *Yap*-knockout (e*Yap*^–/–^) mice exhibit an exacerbated cardiac dysfunction and impaired angiogenesis after MI. WT and e*Yap*^–/–^ mice were subjected to MI or sham surgical operation. (**A**) Cardiac angiogenesis was examined by immunostaining of heart tissue sections with specific anti-CD31 antibody (*n* = 6–8) (scale bar: 50 μm). (**B**) The levels of angiogenetic factors Ang1, VEGF, and VEGFR2 in the myocardium were examined by Western blot (*n* = 4–5). The levels of HSPA12B and YAP in the cytosol (**C**) and nucleus (**D**) in the myocardium were examined by Western blot (*n* = 4–5). β-actin was used as cytosolic loading control and TBP was used as nuclear loading control. (**E** and **F**) Cardiac function was examined by echocardiography 28 days after surgery among WT sham (*n* = 6), WT MI (*n* = 7), e*Yap*^–/–^ sham (*n* = 4), and eYAP^–/–^ MI (*n* = 7) groups. Comparisons of data between groups were made using 1-way ANOVA followed by Tukey’s procedure. *P < 0.05, ^**^P < 0.01, ^***^P < 0.001 compared with indicated groups. MI, myocardial infarction’ YAP, yes-associated protein.

To validate the critical role of endothelial cell YAP in the regulation of angiogenesis after MI, we induced MI in both e*Yap*^–/–^ and WT mice. Endothelial cell–specific *Yap* deficiency was confirmed by immunofluorescence staining (Supplemental Figure 3). We then examined the effects of YAP on cardiac angiogenesis after MI. Figure 8A shows that the positive immunofluorescence staining of CD31 were markedly decreased in the e*Yap*^–/–^ MI hearts compared with WT MI hearts. In addition, the levels of angiogenetic factors such as Ang1, VEGF, and VEGFR2 in the myocardium of e*Yap*^–/–^ MI mice were significantly lower than those in WT MI hearts (Figure 8B). These data demonstrate that endothelial YAP played an important role in the regulation of cardiac angiogenesis after MI challenge.

Because our in vitro data showed that HSPA12B was a target of YAP/TEAD4 (Figure 6A), we examined HSPA12B levels in the myocardium of e*Yap*^–/–^ MI hearts. Figure 8, C and D, shows that HSPA12B expression and the nuclear translocation in the myocardium were markedly suppressed in e*Yap*^–/–^ MI mice compared with WT MI mice, indicating that YAP was needed for HSPA12B expression after MI.

### Endothelial cell–specific deficiency of either HSPA12B or YAP resulted in worsened cardiac function after MI.

Angiogenesis is an essential reparative event after MI, which increases perfusion of the ischemic myocardium and subsequently improves cardiac function (4, 7). Based on these results, cardiac function of WT and e*Hspa12b*^–/–^ mice was examined by echocardiography 28 days after MI surgery. Figure 7, E and F, shows that MI significantly decreased cardiac function in both WT and e*Hspa12b*^–/–^ mice. However, the values for ejection fraction (EF%) (36.7% ± 2.36) and fractional shortening (FS%) (17.1% ± 1.20) in e*Hspa12b*^–/–^ MI mice were markedly lower than those in WT MI mice (46.6% ± 5.67 and 22.7% ± 3.40). We also traced the development of cardiac dysfunction on days 7 and 14 after surgery (Supplemental Figure 4) and found that knockout of e*Hspa12b* exacerbated cardiac dysfunction 14 days after MI. In addition, cardiac fibrosis in the myocardium of e*Hspa12b*^–/–^ MI mice was significantly greater than that in WT MI hearts (Supplemental Figure 5). Next, we examined the effect of endothelial *Yap* deficiency on cardiac function after MI. As expected, *Yap* deficiency led to worsened cardiac dysfunction after MI, compared with WT MI mice, both 14 and 28 days after surgery (Figure 8, E and F; and Supplemental Figure 4). Specifically, 28 days after surgery, the values of EF% (38.0% ± 4.26) and FS% (17.9% ± 2.27) in e*Yap*^–/–^ MI mice were markedly lower than those in WT MI mice (46.0% ± 4.41; 22.3% ± 2.42). Our data suggest that both endothelial cell HSPA12B and YAP were required for maintaining cardiac function, which are associated with angiogenesis.

## Discussion

The present study reveals a mechanism by which endothelial cell HSPA12B regulated cardiac angiogenesis after MI. We demonstrated that endothelial cell HSA12B was translocated into the nucleus accompanied by YAP activation and nuclear translocation in endothelial cells in both in vivo and in vitro studies. Interestingly, we discovered that HSPA12B and YAP cooperatively regulated endothelial cell proliferation and angiogenesis after hypoxic challenge or MI. Of note, HSPA12B was a target gene of YAP and YAP was needed for HSPA12B-promoted angiogenesis. On the other hand, YAP-targeted HSPA12B worked as a transcriptional coactivator to induce YAP activation and nuclear translocation in the regulation of endothelial cell proliferation, migration, and angiogenesis after hypoxia.

Angiogenesis, a target for the development of therapeutic approach to treat ischemic myocardium, plays a key role in improving cardiac function after MI by improving revascularization and blood flow in the long-term left ventricular remodeling (4, 40). Recently, HSPA12B promotes angiogenesis by mediating the turnover of antiangiogenic/protight junction proteins such as AKAP12 (41) and via activating PI3K/Akt signaling (12, 14). Previous studies show that activation of eNOS also contributes to HSPA12B-induced angiogenesis (11, 15). Ma et al. report that overexpression of HSPA12B increases Ang1 and VEGF expression that facilitates tumorigenesis in lung cancer (41). In the present study, we observe that HSPA12B was translocated into the nucleus and promoted endothelial cell proliferation, migration, and angiogenesis after hypoxia. Our observation indicates a potential mechanism by which endothelial HSPA12B regulated angiogenesis.

A recent study shows that HSP27 is required for YAP activation and nuclear translocation in cancer cells (34). Ye et al. report that inhibition of HSP90 inactivates YAP, thus suppressing lung adenocarcinoma cell growth and invasion (33). Collectively, HSPs may participate in the activation of Hippo/YAP signaling pathway. Interestingly, we observe that HSPA12B nuclear translocation was accompanied with YAP activation and nuclear translocation. The Hippo/YAP pathway is a critical player in facilitating cell growth, migration, and differentiation (42, 43). YAP nuclear translocation is required for blood vessel branching and stabilization (44). Importantly, we observe that YAP inhibition attenuated HSPA12B-promoted endothelial cell proliferation and angiogenesis, demonstrating that YAP participates in the HSPA12B-promoted angiogenesis. To investigate the mechanisms by which HSPA12B and YAP work together for the regulation of angiogenesis, we performed a series of experiments using gain- and-loss-of-function approaches. We found that YAP was required for HSPA12B expression and the nuclear translocation, whereas HSPA12B was involved in promoting YAP activation and nuclear translocation, which are associated with endothelial cell proliferation and angiogenesis. Our findings reveal a critical role for endothelial cell HSPA12B in the regulation of angiogenesis, i.e., endothelial HSPA12B and YAP cooperatively regulated angiogenesis. Interestingly, there was an interaction between YAP and HSPA12B in endothelial cells after hypoxia, as evidenced by showing that YAP was found in the HSPA12B immunoprecipitate. It is possible that the association of YAP and HSPA12B was required to facilitate YAP/HSPA12B activation and nuclear translocation for the angiogenesis.

To further investigate the mechanisms by which YAP and HSPA12B cooperatively regulated angiogenesis, we performed ChIP assay and found that HSPA12B was a target gene of YAP/TEAD4 and the binding site was located at the end of the *HSPA12B* gene. Galli et al. claim that YAP and TEAD binding is restricted to distal elements in the genome (45), which is consistent with our observation. A large fraction of the distal elements of TEAD overlapped with H3K27ac active enhancer mark (46), which further confirms the possibility of binding of TEAD4 and *HSPA12B* gene. Additionally, we reveal an important role of HSPA12B in the regulation of YAP expression. Our data suggest 2 possible mechanisms by which HSPA12B regulated YAP expression. First, HSPA12B formed a cluster with YAP/TEAD4 and positively activated YAP at transcriptional level after hypoxia to induce endothelial cell angiogenesis. Second, HSPA12B may have stabilized YAP and abrogated YAP degradation. However, HUVECs were employed for our in vitro experiments. These cells may have subtle differences from cardiac vascular endothelial cells.

To understand the role of endothelial cell HSPA12B and YAP in the regulation of angiogenesis in vivo, we induced MI in WT and endothelial cell–specific *Hspa12b* deficient (e*Hspa12b*^–/–^) or YAP (e*Yap*^–/–^) mice and examined cardiac angiogenesis 28 days after MI. We observed that deficiency of either *Hspa12b* or *Yap* in endothelial cells resulted in significantly impaired cardiac angiogenesis. The levels of angiogenetic factors such as VEGF, Ang1 and VEGFR2 in the myocardium of e*Hspa12b*^–/–^or e*Yap*^–/–^ MI were markedly decreased compared with WT MI hearts. Interestingly, YAP expression and nuclear translocation were markedly increased in WT MI hearts but not the myocardium of e*Hspa12b*^–/–^ MI mice. Similarly, increased HSPA12B expression and nuclear translocation were observed in the myocardium of WT MI mice but not e*Yap*^–/–^ MI hearts. Our in vivo data suggest that endothelial cell HSPA12B and YAP could have cooperatively regulated angiogenesis after MI. In addition, compared with WT mice, e*Hspa12b*^–/–^ MI myocardium showed an increased area of fibrotic scar, suggesting that HSPA12B played an important role in regulating infarct size, thereby regulating cardiac function. Indeed, deficiency of either *Hspa12b* or *Yap* in endothelial cells resulted in worsened cardiac dysfunction after MI, indicating that endothelial cell HSPA12B and YAP served as a protective role in MI-induced cardiac dysfunction.

The present study reveals an important role of endothelial cell HSPA12B in the regulation of angiogenesis by cooperation with YAP after MI. Importantly, we discovered that *HSPA12B* was a direct YAP/TEAD4 target gene and HSPA12B protein functioned as a coactivator for YAP-dependent angiogenesis regulation during MI.

## Methods

### Experimental animals.

Endothelial *Hspa12b*-knockout (e*Hspa12b*^–/–^) mice and endothelial YAP-knockout (e*Yap*^–/–^) mice were generated as described below. WT C57BL/6 mice were purchased from Jackson Laboratory. e*Hspa12b*^–/–^, e*Yap*^–/–^, and WT mice were maintained and bred at the Division of Laboratory Animal Resources at ETSU.

### Generation of endothelial cell–specific HSPA12B and YAP-knockout mice.

The strategy and conformation for endothelial cell–specific *Hspa12b*-knockout mice were described in our recent study by cross-breeding the conditionally targeted *Hspa12b* mice with C57BL/6.Cg-Tg (Tek-cre) strain, which carries Cre recombinase under the control of the Tek promoter (47).

Endothelial cell–specific *Yap*-knockout mice were generated by cross-breeding of *Yap^fl/fl^* mice (a gift from Eric Olson, University of Texas Southwestern Medical Center, Dallas, Texas, USA) with Tek-Cre mice (008863, Jackson Laboratory). Genotypes for the endothelial cell–specific deletion of *Yap* were confirmed by PCR analysis of floxed allele (YAP LA F, ACATGTAGGTCTGCATGCCAGAGGAGG; YAP EX R, AGGCTGAGACAGGAGGATCTCTGTGAG; 600 bp for LoxP allele and 457 bp for WT allele), YAP deletion (YAP LA F, ACATGTAGGTCTGCATGCCAGAGGAGG; YAP SA R, TGGTTGAGACAGCGTGCACTATGGAGC; 338 bp product for deletion). Cre gene expression was also examined by PCR. In addition, immunofluorescence staining was performed to identify endothelial cell–specific deficiency of *Yap*.

### Induction of MI.

MI was induced as previously described (11, 48). Briefly, mice (28–30 g) were anesthetized by 5.0% isoflurane, intubated, and ventilated with room air using a rodent ventilator. Anesthesia was maintained by inhalation of 1.5%–2% isoflurane driven by 100% oxygen flow. Body temperature was regulated at 37˚C by heating pad. After the skin incision, the hearts were exposed through a left thoracotomy in the fourth intercostal space. The left anterior descending coronary artery was permanently ligated with an 8-0 silk ligature. The skin was closed, anesthesia was discontinued, and the animals were allowed to recover in prewarmed cages.

### Echocardiography.

To measure cardiac function, echocardiography was performed on anesthetized mice 28 days after MI as described in our previous studies (48, 49). M-mode tracings were used to measure left ventricular wall thickness, left ventricular end-systolic diameter, and left ventricular end-diastolic diameter. Percentages of FS% and EF% were calculated as previously described (49, 50).

### Histology and immunofluorescence.

Twenty-eight days after MI, hearts were harvested and cut horizontally. One slice below the ligation site was immersion-fixed in 4% buffered paraformaldehyde, embedded in paraffin, and cut at a 5-mm thickness. The sections were stained with Trichrome Stain (Masson) Kit (MilliporeSigma) according to the manufacturer’s protocol as previously described (51). For immunofluorescence staining, sections were stained with specific anti-CD31 antibody (1:50 dilution; ab28364, Abcam), anti-YAP antibody (1:100 dilution; 4912s, Cell Signaling Technology), or anti-HSPA12B antibody (9) (1:100 dilution; a gift from Zhihua Han, ETSU, Johnson City, Tennessee, USA). The stained sections were examined using EVOS Microscope (Thermo Fisher Scientific) or Confocal Microscope (Leica) and measured using NIH ImageJ software (version 1.389).

### Endothelial cell culture and transfection.

HUVECs were cultured in Gibco Ham’s F-12K (Kaighn’s) Medium supplemented with growth factors and 5% FBS. When HUVECs reached 70%–80% confluence, they were transfected with siRNA (80 nmol; Invitrogen by Life Technologies) specific for HSPA12B or YAP. Scrambled siRNAs served as controls. The siRNA transfection efficiency was confirmed by siPORT NeoFX Transfection Agent (Invitrogen by Life Technologies). In addition, YAP inhibitor VP (1 mmol/L) was also employed to suppress YAP activation before the cells were subjected to hypoxic challenge. In separate experiments, HUVECs were transfected with adenovirus expressing HSPA12B labeled with GFP (Ad-HSPA12B, MOI = 10). Adenovirus expressing scrambled GFP served as control. Twenty-four hours after transfection, the HUVECs were incubated at 37°C with 5% CO_2_ and 0.1% O_2_ in a hypoxia chamber (Pro-Ox Model C21, BioSpherix) for an additional 24 hours as previously described (52). The cells that were not subjected to hypoxia were incubated at 37°C with 5% CO_2_ and served as controls (normoxia). In some experiments, administration of CHX (100 μg/mL, Sigma) or MG132 (12 μM, Sigma) was performed to inhibit protein synthesis.

### Endothelial cell proliferation assay.

The HUVEC proliferative activity was measured by Edu incorporation into the cells (Click-iT Edu Imaging Kit, Invitrogen by Life Technologies) and MTT assay (Thermo Fisher Scientific) according to the manufacturer’s protocols (53). Cells were seeded in 24-well plates for Edu incorporation assay and 96-well plates for MTT assay. The proliferation rate of Edu incorporation was calculated by normalizing the number of Edu-positive cells to the DAPI-stained cells.

### Endothelial cell migration assay.

Endothelial cell migration capacity was measured by the scratch (or wound-healing) assay (54) 24 hours after siRNA or Ad-HSPA12B transfection using 12-well plates. HUVECs were scratched with 200-μl tips when cells reached 80% confluence, incubated with empty F-12K medium without growth factor after scratching, and photographed 24 hours after injury. Mitomycin C (5 μg/mL; Invitrogen) was used to inhibit cell proliferation 1 hour before scratch. The percentage closure of the wound was analyzed by an image analyzer (NIH ImageJ software).

### Matrigel-based in vitro angiogenesis assay.

Endothelial cell angiogenesis was assessed using Matrigel-based angiogenesis assay (9). Briefly, HUVECs were seeded on Matrigel-coated 96-well plates (Corning) with 10^4^ cells/well and photographed 6 hours after incubating at 37°C with 5% CO_2_. Total number of master junction was quantified by NIH ImageJ software.

### Western blotting.

Western blotting was performed as previously described (48, 49). Briefly, tissue or cellular proteins were extracted from ischemic hearts or cells. Protein concentration was determined by BCA Protein Assay Kit (Thermo Fisher Scientific). The cellular proteins were separated by SDS-PAGE and transferred onto Hybond ECL membranes (Amersham Pharmacia). The ECL membranes were incubated with the appropriate primary antibodies (anti-Angiopoietin1, 1:200 dilution, sc-6320, Santa Cruz Biotechnology; anti-VEGF, 1:1000 dilution, ab46154, Abcam; anti-VEGFR2, 1:1000 dilution, 2479s, Cell Signaling Technology; anti-YAP, 1:1000 dilution, 4912s, Cell Signaling Technology; anti-GAPDH, 1:1000 dilution, 2118s, Cell Signaling Technology; anti-β-actin, 1:1000 dilution, 3700s, Cell Signaling Technology; anti-Histone3, 1:2000 dilution, nb500-171, Novus Biologicals; anti-TBP, 1:1000 dilution, ab51841, Abcam; HSPA12B antibody, 1:1000 dilution) followed by incubation with peroxidase-conjugated secondary antibodies (7074s and 7076s, Cell Signaling Technology) and analysis by the ECL system (Amersham Pharmacia). The signals were quantified using the G:BOX gel imaging system (Syngene).

### Immunoprecipitation.

Immunoprecipitation was performed as previously described (22). Briefly, we seeded cells in 100-mm dishes and about 200 μg of total cellular proteins were incubated with 2 μg of anti-HSPA12B, anti-TEAD4 (ab58310, Abcam) or anti-YAP antibodies for 12 hours at 4°C followed by adding protein A/G-agarose beads (20 μl; Santa Cruz Biotechnology). The precipitates were washed 4 times with lysis buffer and boiled in SDS sample buffer. The supernatant was subjected to immunoblotting with appropriate antibodies.

### qRT-PCR.

Total RNA was isolated from heart tissues or cultured cells using RNAzol RT (Molecular Research Center) in accordance with the manufacturer’s protocol as previously described (53). qRT-PCR was conducted using a 4800 RT-PCR machine (Bio-Rad). mRNA was reversed to cDNA by cDNA Reverse Kit (Applied Biosystems). qRT-PCR was performed using specific TaqMan primers (Applied Biosystems) and TaqMan Universal Master Mix (Applied Biosystems). The mRNA levels of *HSPA12B*, *YAP*, *VEGF*, and *Ang1* were quantified with the 2 (-ΔΔct) relative quantification method that was normalized to *β-Actin* (Applied Biosystems).

### ChIP-qPCR.

ChIP assay was performed according to the manufacturer’s protocol (High-Sensitivity ChIP Kit, ab185913, Abcam). qPCR was performed using SYBR green ReadyMix (MilliporeSigma) and sequences of primers are listed in Supplemental Table 1. qRT-PCR was conducted using a 4800 RT-PCR machine (Bio-Rad).

### Statistics.

Data are expressed as mean ± SD. Comparisons of data between groups were made using 2-tailed *t* test or 1-way ANOVA followed by Tukey’s procedure for multiple range tests. A *P* value of less than 0.05 was considered to be significant.

### Study approval.

All experimental procedures were performed in accordance with the Guide for the Care and Use of Laboratory Animals (National Academies Press, 2011) and approved by the ETSU Committee on Animal Care.

## Author contributions

MF, KY, XW, and CL conceived the study. MF, KY, YW, FT, and TH conducted the experiments and acquired the data. MF and KY analyzed the data. MF and CL wrote the manuscript. KY, XW, TH, LL, DLW, and CL reviewed the manuscript.

## Supplementary Material

Supplemental data

## Version 1 08/13/2020

In-Press Preview

## Version 2 09/17/2020

Electronic publication
