# Supplementary material for: Endothelial cell HSPA12B and yes-associated protein cooperatively regulate angiogenesis following myocardial infarction
Source: JCI Insight. 2020 Sep 17;5(18):e139640. doi: 10.1172/jci.insight.139640 (PMC7526558; doi:10.1172/jci.insight.139640)
Supplement: Supplemental data [file jciinsight-5-139640-s169.pdf]

Supplemental Figures

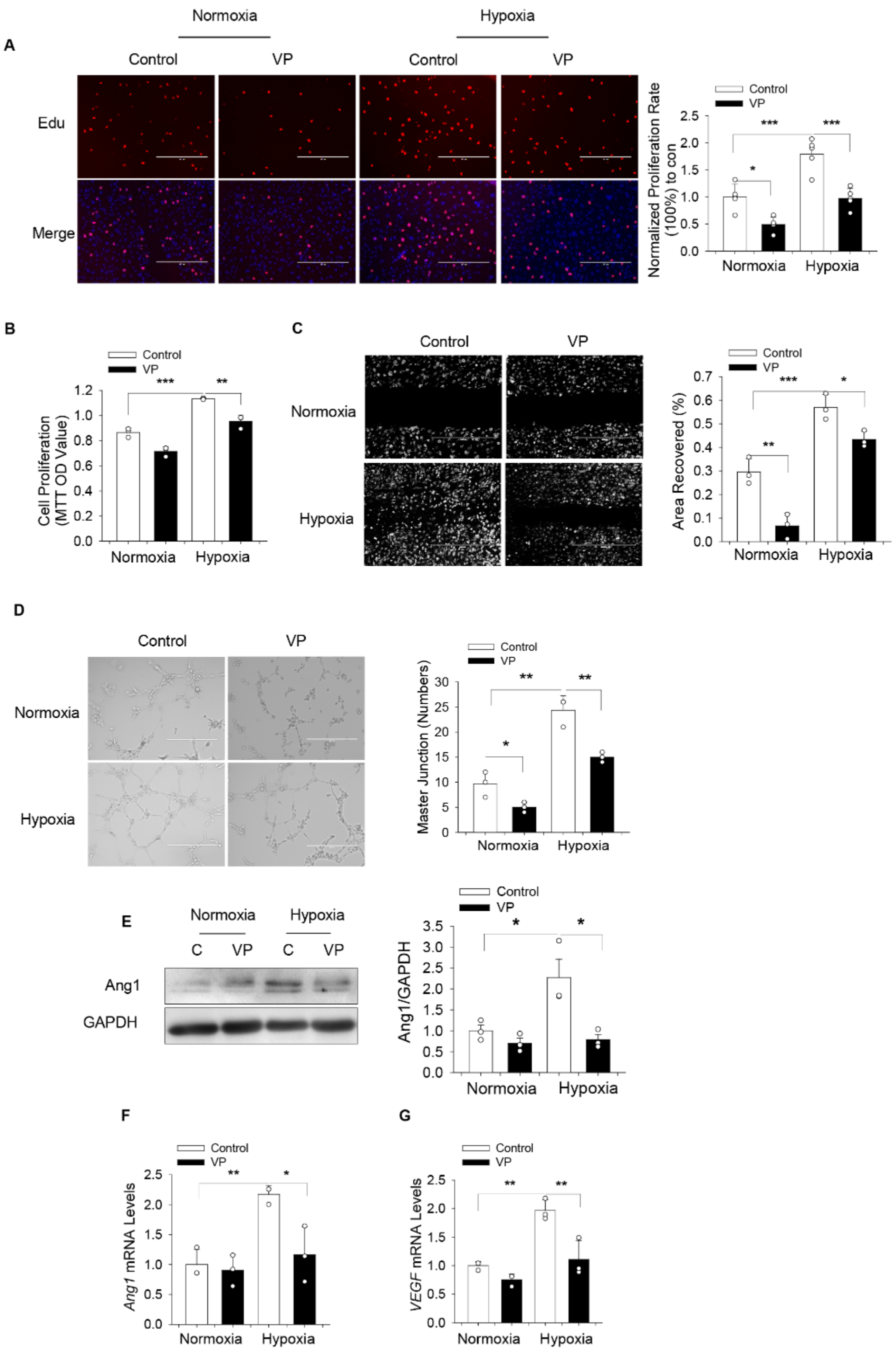

Supplemental Figure 1. YAP inhibition suppresses hypoxia-induced endothelial cell proliferation, migration, and angiogenesis. Endothelial cells were treated with YAP inhibitor, Verteporfin (VP, 1 mM/L) before the cells were subjected to hypoxic challenge. Endothelial cell proliferation was determined by Edu incorporation (**A**) and MTT assay (**B**), scale bars: 400μm. **C**, Cell migration was determined by wound-healing assay, scale bars: 400μm. **D**, Angiogenesis was examined by Matrigel assay, scale bars: 400μm. Expression of *Ang1* and *VEGF* were examined by Western blot (**E**) and qRT-PCR (**F-G**). n=3-5 independent experiments/group. Comparisons of data between groups were made using one-way ANOVA followed by Tukey's procedure. * $P < 0.05$, ** $P < 0.01$, *** $P < 0.001$ compared with indicated group.

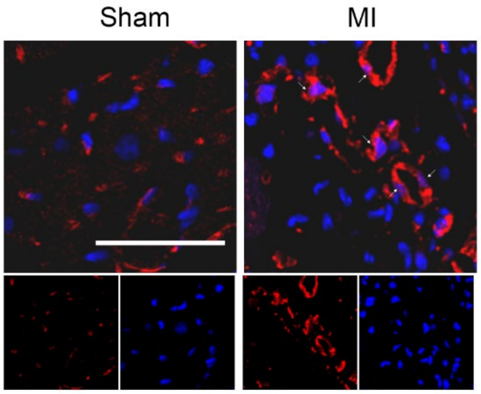

Supplemental Figure 2. MI induced HSPA12B expression and nuclear translocation. HSPA12B nuclear localization was examined by immunofluorescent staining with anti-HSPA12B antibody (red). Nucleus were stained with DAPI (blue). n=3/group. Scale bar: 100μm.

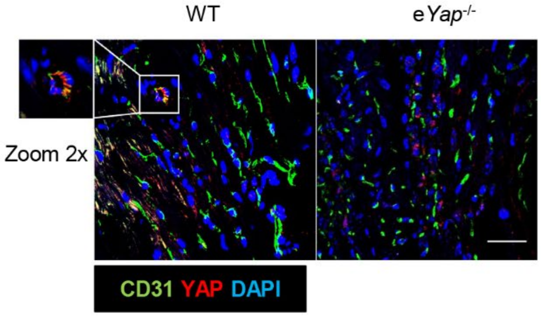

Supplemental Figure 3. Immunofluorescent staining of YAP expression in heart tissue sections. n=3/group. Scale bar: 50μm.

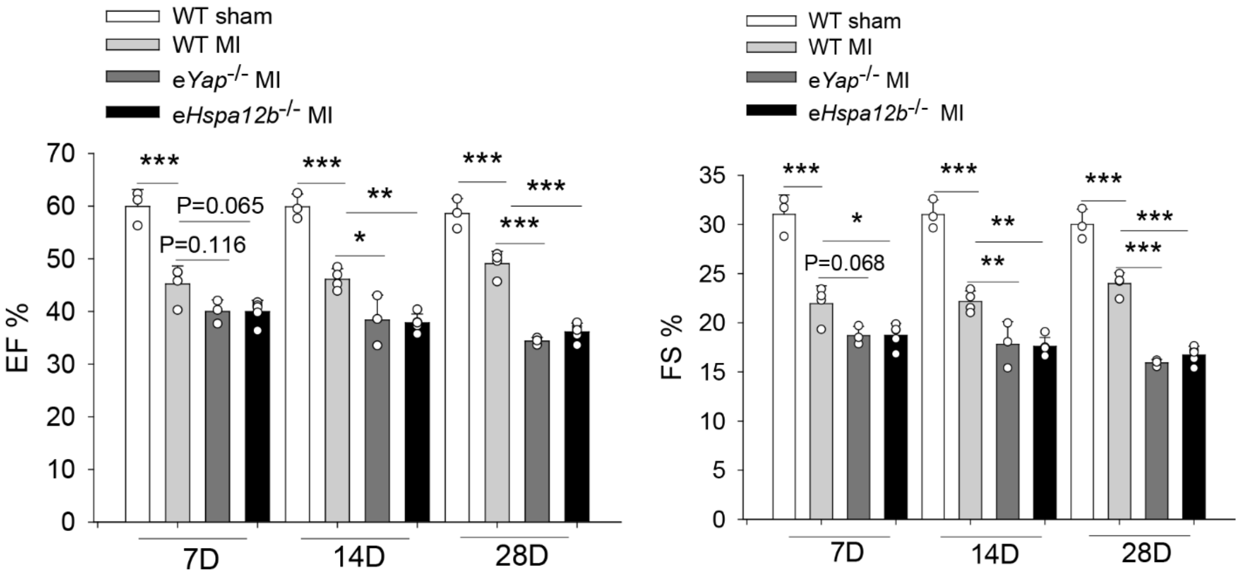

Supplemental Figure 4. Endothelial specific *Hspa12b* (e*Hspa12b*$^{-/-}$) or *Yap* (e*Yap*$^{-/-}$) knockout mice exhibit an exacerbated cardiac dysfunction after MI. Cardiac function was examined by echocardiography 7, 14 and 28 days after surgery among WT sham (n=3), WT MI (n=4), e*Yap*$^{-/-}$ MI (n=3) and e*Hspa12b*$^{-/-}$ MI (n=5) groups. Comparisons of data between groups were made using one-way ANOVA followed by Tukey's procedure. * $P< 0.05$, ** $P <0.01$, *** $P <0.001$ compared with indicated group.

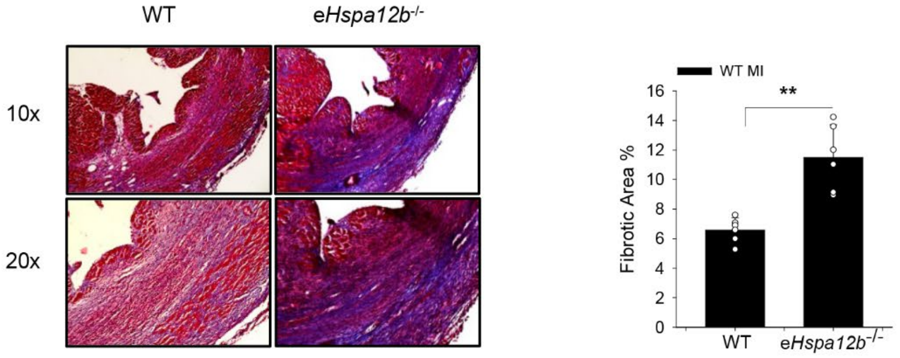

Supplemental Figure 5. Endothelial specific *Hspa12b* knockout (e*Hspa12b*$^{-/-}$) mice exhibit an increased cardiac fibrosis after MI. Cardiac fibrosis was examined by staining of heart tissue sections with Trichrome stain (Masson) kit. n=6/group. Comparison of data between groups was made using t-test. ** $P <0.01$ compared with indicated groups.

Table 1. PCR primers used for ChIP analysis.

Names	Sequences (5'->3')
HSPA12B Forward Primer	TGCTGATATGGCGTGGAGAC
HSPA12B Reverse Primer	CACCCCCTTCTGGTCAGTTC
YAP Forward Primer	GCGGATATGAACATGGCTGCT
YAP Reverse Primer	TGGGCAAAGTTCCTATGCTG
CTGF Forward Primer	AGGCTTTTATACGCTCCGGG
CTGF Reverse Primer	TGAGTGTCAAGGGGTCAGGA
